# 579 Outcomes of an Independent Burn Advanced Practice Provider Peer Review Process

**DOI:** 10.1093/jbcr/irac012.207

**Published:** 2022-03-23

**Authors:** Stephanie Steiner, Lauren Lewis, Kevin Baker, John Crow, Anjay Khandelwal

**Affiliations:** Akron Children’s Hospital, Cuyahoga Falls, Ohio; Paul and Carol David Foundation Burn Institute; Akron Children's Hospital, Akron, Ohio; Akron Children's Hospital, Norton, Ohio; Akron Children's Hospital, Akron, Ohio; Akron Children's Hospital, Akron, Ohio

## Abstract

**Introduction:**

Burn peer review (PR) is an important process for quality assurance and process improvement. Our division consists of five burn surgeons, seven advanced practice providers (APPs) and four night house officer (NHO) APPs. As the APPs are intimately integrated within the burn service - both on the inpatient (IP) and outpatient (OP) settings, a subsequent need to create an independent peer review process for the APPs was identified. We describe our process and evaluate the results after two years of a monthly APP PR meeting.

**Methods:**

All data is entered into a quality management software. Triggers for APP PR include complex discharge, inappropriate OP referral, delay in treatment, OP treatment greater than three weeks without surgical intervention, pressure wound, patient discharge with greater than 30 morphine equivalent dose (MED) of narcotic, readmission or unexpected admission from OP clinic, substance abuse, and self referral. Cases with individual improvement opportunities result in real time direct feedback from the director, lead APP, and peers. These may be escalated to the provider’s ongoing professional performance evaluation (OPPE) or trigger a focused professional performance evaluation (FPPE). Systemic issues identified are referred for multidisciplinary discussion in our Performance Improvement/Quality Assurance (PI/QA) process. Complicated issues involving multiple disciplines may trigger a root cause analysis. If an individual or system issue is not identified, a group discussion about process improvement and standardization of practice ensues.

**Results:**

From initiation of the Burn APP PR process in May of 2019, to September 2021, there were 37 cases reviewed. Twelve were for indicated triggers and 25 for self-referrals. Breakdown of these cases is as follows: outpatient treatment greater than three weeks without surgical intervention (3), readmission or unexpected admission from outpatient clinic (4), delay in treatment impacting clinic course (5). Self referred cases included graft loss (8), cellulitis (3), venous thromboembolic event (2), and wound dressing discussion (2). There were three cases where clinical issues were identified of which one was referred for root cause analysis, and the other two led to real-time education and feedback from the director.

**Conclusions:**

PR is important to ensure that high quality patient care standards are met. Autonomous APP practice is important for the function of a team, quality of care delivered, professional satisfaction of the APP, and enables financial optimization for burn centers. Creating an independent burn APP peer review process allows for time-sensitive feedback to patient care related events, improves collaboration and practice standardization, and provides an important educational opportunity for the team.

